# Modulation in Biofertilization and Biofortification of Wheat Crop by Inoculation of Zinc-Solubilizing Rhizobacteria

**DOI:** 10.3389/fpls.2022.777771

**Published:** 2022-02-25

**Authors:** Ramesh Chandra Yadav, Sushil K. Sharma, Ajit Varma, Mahendra Vikram Singh Rajawat, Mohammad Shavez Khan, Pawan K. Sharma, Deepti Malviya, Udai B. Singh, Jai P. Rai, Anil K. Saxena

**Affiliations:** ^1^Amity Institute of Microbial Technology, Amity University, Noida, India; ^2^Plant-Microbe Interaction and Rhizosphere Biology Lab, ICAR-National Bureau of Agriculturally Important Microorganisms, Kushmaur, India; ^3^Department of Mycology and Plant Pathology, Institute of Agricultural Sciences, Banaras Hindu University, Varanasi, India

**Keywords:** rhizobacteria, wheat, zinc phosphate, biofortification, zinc-solubilizing rhizobacteria

## Abstract

Zinc is an important micronutrient needed for the optimum growth and development of plants. Contrary to chemical zinc fertilizers, the use of zinc-solubilizing bacteria is an environmentally friendly option for zinc enrichment in edible parts of crops. This study was conducted with the objective of selecting potential zinc-solubilizing rhizobacteria from the rhizosphere of chickpea grown in soils of eastern Uttar Pradesh and further assessing their impact on the magnitude of zinc assimilation in wheat crops. Among 15 isolates, CRS-9, CRS-17, CRS-30, and CRS-38 produced net soluble zinc in broth to the tune of 6.1, 5.9, 5.63, and 5.6 μg ml^–1^, respectively, in zinc phosphate with the corresponding pH of 4.48, 5.31, 5.2, and 4.76. However, the bacterial strains CRS-17, CRS-30, CRS-38, and CRS-9 showed maximum zinc phosphate solubilization efficiency of 427.79, 317.39, 253.57, and 237.04%, respectively. The four bacterial isolates were identified as *Bacillus glycinifermentans* CRS-9, *Microbacterium oxydans* CRS-17, *Paenarthrobacter nicotinovorans* CRS-30, and *Bacillus tequilensis* CRS-38 on the basis of morphological and biochemical studies and 16S rRNA gene sequencing. Bacterial inoculants significantly colonized the roots of wheat plants and formed a biofilm in the root matrix. These strains significantly increased seed germination (%) and vigor indices in wheat grown under glasshouse conditions. After 30 days of sowing of wheat under microcosm conditions, eight zinc transporter (*TaZIP*) genes were expressed maximally in roots, with concomitant accumulation of higher zinc content in the bacterially treated plant compared to the absolute control. Out of the four strains tested, two bacteria, *B. tequilensis* CRS-38 and *P. nicotinovorans* CRS-30, improved seed germination (%), vigor indices (2–2.5 folds), plant biomass, grain yield (2.39 g plant^–1^), and biofortificated grains (54.25 μg g^–1^Zn) of wheat. To the best of our knowledge, this may be the first report on the presence of zinc solubilization trait in *B. glycinifermentans* CRS-9, *M. oxydans* CRS-17, and *P. nicotinovorans* CRS-30.

## Introduction

The most important challenge to researchers, scientists, and policymakers is to ensure food and nutritional security for the burgeoning population, which may reach 9.8 billion by the year 2050. Food security entails increasing agricultural productivity and improvement in product quality while reducing the adverse footprints of cultivation practices in natural resources and the environment. Among the 17 sustainable development goals (SDGs), SDGs 1–3 are directly related to sustainable agricultural growth and aim to eliminate poverty and hunger, and ensure good health and well-being, respectively. More than half of the global population suffers from micronutrient malnutrition in developed and developing countries (Bouis, 2003). Among various micronutrients, zinc (Zn) is required for the optimal growth and development of all the organisms (Hafeez et al., 2013). In plants, Zn plays an important role in carbohydrate and auxin metabolism (Alloway, 2004, 2008a,b), protein synthesis, and assimilation of other major nutrients, and it also acts as a significant antioxidant (Singh et al., 2018c). Currently, Zn deficiency is common in plants, human beings, and animals. Zn deficiency in plants causes a reduction in photosynthesis, flowering and fruit development, synthesis of carbohydrate and phytohormones, shoot and root development, and leaf size; it induces chlorosis and susceptibility to heat, light, and fungal infections; it affects water uptake and delays crop maturity, leading to decrease in crop yield and nutritional quality of grains (Alloway, 2004; Tavallali et al., 2010). Zinc deficiency results in yellowing of leaves and stunted growth in wheat plants. Zinc deficiency in humans is due to consumption of zinc-deficient food including wheat grown in Zn-deficient soils. Globally, the widespread occurrence of Zn-deficiency in crops is due to the low solubility of Zn rather than low Zn availability in soil (Iqbal et al., 2010). More than 50% of Indian soils have been estimated to be deficient in Zn (Ramesh et al., 2014a). The total content of zinc in soil ranges from 50–300 μg g^–1^ soil. Cereal crops grown on these soils are zinc-deficient. Urgent corrective measures are required to eliminate Zn deficiency in edible crops to ensure proper Zn nutrition.

In general, plants have several mechanisms to overcome Zn deficiency. Among them, Zn finger transcription factors are important for the development of floral tissues, flowering, fertilization, and fruiting (Epstein and Bloom, 2005). Zn also plays a vital role in the regulation of uptake and translocation of Zn in plants and modulates several biological pathways during environmental stress. Furthermore, genetic biofortification is time-consuming, and it may not be successful in some different soil environments (Cakmak, 2008). The agronomic strategy of biofortification using zinc as a fertilizer, crop rotation (White and Broadley, 2009; Dimpka and Bindraban, 2016), and application of organic manures (Aghili et al., 2014; Helfenstein et al., 2016) and microbial inoculants (Saravanan et al., 2007a,b; Ramesh et al., 2014b; Wang et al., 2014; Gandhi and Muralidharan, 2016) are gaining momentum. In this context, using plant growth-promoting rhizobacteria (PGPR) with zinc-solubilizing ability as rhizobacterial inoculants is a cost-effective and eco-friendly alternative for zinc biofertilization. Zinc solubilizing bacteria alone or with organic materials may enhance the bioavailability of native and applied zinc in plants through different mechanisms.

Several plant growth-promoting microorganisms (PGPMs) including rhizobacteria have been reported to be effective zinc solubilizers (Fasim et al., 2002; Saravanan et al., 2007a; Abaid Ullah et al., 2015; Singh et al., 2018a,b). These bacteria colonize the rhizosphere and intensify zinc bioavailability by solubilizing complex zinc compounds, thereby improving plant growth and development. Bacteria are endowed with various zinc-solubilizing mechanisms, and acidification is one of the key processes (Ramesh et al., 2014a; Hussain et al., 2015; Naz et al., 2016; Yadav et al., 2020). In soil, bacteria produce organic acids, which sequester zinc cations, resulting in a reduction in pH of nearby soil. Moreover, anions are able to chelate zinc and increase the solubility of zinc (Jones and Darrah, 1994; Yadav et al., 2020). Solubilization probably also takes place through other mechanisms, viz. production of siderophores and proton, oxido-reductive systems in cell membranes, and chelated ligands (Wakatsuki, 1995; Chang et al., 2005; Saravanan et al., 2011). Moreover, several bacilli have been reported to cause modulation of growth- and yield-contributing factors, along with zinc biofortification of wheat and soybean crops (Sharma et al., 2012; Khande et al., 2017; Yadav et al., 2020). Globally, wheat (*Triticum aestivum* L.) is an important crop, being a source of food, feed, fiber, and fuel (Singh et al., 2019a,b). As against cereals like rye and barley, the importance of bread wheat in human nutrition has jumped up because of changes in its use over the last two millennia, and this has led to an exponential increase in wheat production worldwide including in India. In general, wheat contains 75–80% carbohydrates, 9–18% protein, fiber, many vitamins (especially B group of vitamins), calcium, iron, and many other macro- and micro-nutrients (Igrejas and Branlard, 2020). In the Indian subcontinent, more than one billion people depend on diets containing cereals (rice and wheat), pulses, and oilseeds that are deficient in Zn (Prasad et al., 2010). Keeping the significance of zinc biofortification of wheat and possible role of Zn-solubilizing rhizobacteria in view, this investigation aimed to characterize potential zinc solubilizing rhizobacterial isolates from chickpea rhizosphere and their application in zinc biofortification of wheat crop. Rhizobacteria isolated from chickpea rhizosphere were used in this study, with the assumption that these bacteria are also associated with the wheat crop, because farmers grow either chickpea or wheat crop in the same piece of land during the *Rabi* season (December to April).

## Materials and Methods

### Chemicals, Bacterial Strains, and Cultural Conditions

General media, glucose, zinc oxide, zinc carbonate, zinc phosphate, hydrogen peroxide, nicotinamide adenine dinucleotide, etc., were purchased from HiMedia Pvt., Ltd., Mumbai (India). Analytical-grade chemicals and standards were procured from Sigma–Aldrich (St. Louis, MO, United States). Bovine serum albumin and high-performance liquid chromatography (HPLC)-grade solvents and chemicals were procured from E. Merck (Mumbai, India), while molecular-grade chemicals were purchased from Banglore GeNi (Bangaluru, India), BioRAD (Gurugram, Haryana, India), and Agilent (Mumbai, India). PCR and qPCR primers were synthesized from Eurofin Private Limited, Bangaluru (India).

In this study, 15 rhizobacterial isolates were retrieved from the rhizosphere of chickpea grown in different agroclimatic regions of eastern Uttar Pradesh, India (Supplementary Table 1). These isolates were designated as CRS-9 (CRS–chickpea rhizosphere soil), CRS-17, CRS-26, CRS-30, CRS-37, CRS-38, CRS-42, CRS-43, CRS-45, CRS-47, CRS-50, CRS-54, CRS-55, CRS-57, and CRS-77, and cultured in a nutrient broth medium at 28*^o^*C for 28–48 h with shaking at 120 rpm and preserved in 20% glycerol stock at –80*^o^*C in deep freezer.

### Selection of Potential Zinc-Solubilizing Rhizobacteria

Screening of the fifteen (15) rhizobacterial isolates was performed for zinc solubilization using a tris-minimal agar medium supplemented with D-glucose and with different insoluble zinc compounds as per the methods of Fasim et al. (2002), with slight modification (Sharma et al., 2011). Specifically, the tris-minimal medium was amended separately with zinc oxide [ZnO] (1.244 g l^–1^), zinc phosphate [Zn_3_ (PO_4_)_2_] (1.9882 g l^–1^), and zinc carbonate [ZnCO_3_.2H_2_O] (1.728 g l^–1^), equivalent to the concentration of 0.1% Zn as sole zinc source to find out the ability of rhizobacterial isolates to solubilize zinc oxide, zinc phosphate, and zinc carbonate. After autoclaving (at 121*^o^*C for 15 min), the culture medium was transferred to sterilized Petri plates. Freshly grown bacterial cultures were spot-inoculated in triplicates in the medium using sterile toothpicks, followed by incubation of the plates at 28°C for 7 days in the dark to observe Zn solubilization in the form of a clear halo zone around colonies. After 7 days, colony diameter and the diameter of the halo zone (mm) formed around the colony were measured. Afterward, the same plates were flooded with a methyl red solution to observe acid production by bacteria. Change in color of a clear zone from yellow to red following methyl red solution indicates a positive reaction (Garcia and Isenberg, 2007). The extent of zinc solubilization by different isolates was ensured by measuring the zone of solubilization around the colonies. Zinc solubilization index (ZSI) and zinc solubilization efficiency (ZSE) of the isolates were determined (Vazquez et al., 2000; Ramesh et al., 2014b). Isolates showing maximum ZSE were considered to be potential zinc solubilizers.

### Quantitative Assay for Zinc Solubilization

Potential zinc solubilizing rhizobacterial isolates were further tested for their ability to release soluble zinc in a liquid medium and their effect on the pH of the medium. The isolates were inoculated in a tris-minimal broth medium supplemented with 0.1% Zn as zinc phosphate. For each isolate, 50 ml of the liquid tris-minimal broth medium supplemented with 0.1% Zn as zinc phosphate was taken in a 100-ml Erlenmeyer flask and autoclaved. One ml aliquot of each culture with a cell load of 10^8^cfu/ml was inoculated in each flask. A tris-minimal broth medium supplemented with zinc phosphate but without bacterial inoculation served as the absolute control. Each treatment was replicated thrice. All the flasks were incubated at 28*^o^*C in an orbital shaker at 120 rpm for 10 days. After 10 days of incubation, samples were withdrawn, and aliquots of the liquid broth medium were centrifuged at 10,000 rpm for 8–10 min to remove cell debris, and a clear supernatant was collected and fed directly to an atomic absorption spectrophotometer for determination of released soluble zinc (μg Zn ml^–1)^ in the clear broth, and the pH of each culture and the uninoculated broth was measured (Ramesh et al., 2014b).

### Analysis of Gluconic Acid Produced by Zinc-Solubilizing Bacteria Using High-Performance Liquid Chromatography

Gluconic acid is the pre-dominant acid produced during bacterial Zn solubilization. In this study, gluconic acid was quantified using an HPLC system (Separon SGX C18 column; Shimadzu, Kyoto, Japan) equipped with a quaternary pump, an auto-sampler, a DAD detector, and a degasser and according to Larcher et al. (2009) but with slight modifications (Sunithakumari et al., 2016). Specifically, the rhizobacterial isolates were tested for production of gluconic acid. They grew in 50-ml of the tris-minimal broth medium supplemented with 0.1% Zn as zinc phosphate for 10 days. After incubation, the bacterial cultures were centrifuged (12,000 rpm, 20 min, and 4°C), and the supernatant was passed through 0.2-μm membrane filters. A filter-sterilized culture filtrate was collected and analyzed using the HPLC system. Elution was performed with an isocratic flow consisting of acetonitrile:water (30:70v/v) with a flow rate of 1 ml/min at 210 nm using a UV/VIS detector. Retention time was 2.4 min with 10 μl of injection. The detection limit of the instrument is up to 0.5 mg kg^–1^, and standard gluconic acid is detected in the range of 50 mg kg^–1^. Pure gluconic acid was purchased from Sigma-Aldrich (Mumbai, India) and used as a reference. Thus, the gluconic acid present in the culture filtrate was determined by comparing the retention time and peak area of the sample with the standards of gluconic acid.

### Screening of Plant Growth-Promoting Attributes in Zinc-Solubilizing Rhizobacteria

Plant growth promotion attributes of the isolates, such as phosphate solubilization, potash solubilization, production of siderophore, indole-3-acetic acid (IAA), and ammonia, were also assessed (Dinesh et al., 2018). Phosphate solubilization was determined using a Pikovaskya medium containing 0.1% tri-calcium phosphate, and the release of phosphorus by phosphate solubilization was studied by the method described by Olsen and Sommers (as cited in Penrose and Glick, 2003). However, the K solubilization test was performed according to Rajawat et al. (2016). The chrome azurol S (CAS) agar assay described by Schwyn and Neilands (1987) was followed to screen the bacterial isolates for siderophore production. The test isolates were spot inoculated on CAS agar plates and incubated at 30 ± 2°C for 5 days. Development of yellow-orange halo zones around the growing colonies is an indication of siderophore production. IAA production by rhizobacterial isolates was estimated as per the method of Ahmad et al. (2020). Bacterial isolates were grown for 3 days in a Luria Bertani (LB) broth at 28 ± 2°C and were centrifuged at 8,000 rpm for 30 min. The supernatant (2ml) was mixed with two drops of orthophosphoric acid and 4 ml of the Salkowaski reagent, and incubated in the dark at room temperature for 20 min followed by measurement of absorbance at 530 nm using pure IAA (Sigma-Aldrich, United States) as standard. Ammonia content was detected by adding 1 ml of a Nessler reagent to 72-h old cultures grown in peptone broth. Positive samples showed a yellowish-brown color. Bacterial isolates were screened for ACC deaminase production following the method of Bal et al. (2013).

### Characterization of Zinc-Solubilizing Rhizobacteria

Morphological and biochemical characterizations of the four potential zinc-solubilizing isolates were performed as per the methods described in *Bergey’s Manual of Determinative Bacteriology*. Biochemical characterization includes Gram-reaction, catalase, oxidase, nitrate, H_2_S, citrate utilization, Voges Proskauer’s, esculin hydrolysis, methyl red, indole, ONPG, lysine, ornithine, urease, phenylalanine, malonate, arabinose, xylose, adonitol, rhamnose, cellobiose, melibiose, saccharose, raffinose, trehalose, glucose, and lactose utilization. Identification of the potential isolates was performed using 16S rRNA gene sequence similarity as per the method described by Singh et al. (2016). Phylogenetic analysis was performed using Molecular Evolutionary Genetics Analysis (MEGA-X), and 16S rRNA gene sequences were submitted to the NCBI GenBank^1^.

### Evaluation of Zinc Solibilizing Rhizobacteria

#### Planting Materials and Growth Conditions

Microcosm experiments were carried out on wheat (*cv.* HD-2967) as a test crop in a glasshouse situated at Research Farm of ICAR-Indian Institute of Seed Sciences (ICAR-IISS), Mau, Uttar Pradesh, India (25°53′ 56.99″N 83°29′18.29″E, elevation 74 m) during the winter season (2019 and 2020) to investigate the impact of zinc-solubilizing rhizobacteria on wheat growth and development. The wheat seeds used in the experiments were procured at ICAR-IISS, Mau, Uttar Pradesh. The weather conditions during the growing period were: mean temperature 22–25°C, relative humidity of 70–75% with an 11/13-h photoperiod.

#### Preparation of Rhizobacterial Inoculants

Four potential rhizobacterial strains, *Bacillus glycinifermentans* (CRS-9), *Microbacterium oxydans* (CRS-17), *Paenarthrobacter nicotinovorans* (CRS-30), and *Bacillus tequilensis* (CRS-38) were used for the evaluation of their effects on plant growth, rhizosphere properties, zinc acquisition, and mobilization. The bacterial formulations were prepared using a sterile saline solution and employing the methods of Singh et al. (2016). The colony forming unit (CFU) count of the formulations was adjusted to 2 × 10^8^ cfu ml^–1^ at the time of application.

#### Root Colonization and Formation of Biofilm

Bio-primed seeds (20 ml kg^–1^ seeds) were sown in small pots (4 in^2^ × 5 in^2^) containing a sterile soil mixture (sand:soil:vermiculite in 1:1:1 ratio) under glasshouse conditions. After 15 days of sowing, plants were uprooted gently and washed in running tap water. Root samples were fixed with an osmium tetraoxide solution (HiMedia) and 2.5% glutaraldehyde (HiMedia) according to Singh et al. (2021). After fixation, the roots were dehydrated using a gradient of ethyl alcohol (5, 10, 20, 50, 70, 90, and 100%) and dried under vacuum. Thereafter, the samples were coated with gold (20 nm) and visualized under a scanning electron microscope (S-3400N; Hitachi, Chiyoda-ku, Tokyo, Japan).

#### Effects on Seed Germination and Vigor Indices

Effects of seed inoculation on germination and vigor indices of the plants grown in pots after 15 and 30 days of sowing, respectively, were recorded. To see the effects of seed inoculation on germination and vigor indices, seeds were surface-sterilized (1% NaOCl for 60 s) and subsequently rinsed with sterile distilled water thrice to remove NaOCl. The seeds were bioprimed (20 ml kg^–1^ seeds), incubated overnight at 28°C, and sown in pots containing experimental soil under glasshouse conditions. For the germination test, a set of experiments was conducted following the protocols of the International Seed Testing Association (ISTA, 2003; Singh et al., 2016). Specifically, for the seed germination test, 500 seeds were taken and sown in pots (20 seeds pot^–1^) containing a sterile soil mixture. The germinated seeds were counted after 15 days of sowing, and the percent seed germination was calculated. To see the effects of seed inoculation on vigor indices (vigor indexes I and II), bioprimed seeds were sown in pots (5 seeds pot^–1^) under glasshouse conditions. After 30 days of sowing, vigor indexes I and II were calculated according to Singh et al. (2016).

#### Effects of Seed Inoculation on Plant Growth, Yield, and Zn Content

##### Experimental Setup

The glasshouse experiment was laid out with six treatments and 10 replications each in a complete randomized block design (CRBD). Treatments were: T_1_: the absolute control (without zinc phosphate + without bacteria), T_2_: Zn_3_ (PO_4_)_2_ (without bacteria), T_3_: Zn_3_ (PO_4_)_2_ + CRS-9, T_4_: Zn_3_ (PO_4_)_2_ + CRS-17, T_5_: Zn_3_ (PO_4_)_2_ + CRS-30, and T_6_: Zn_3_ (PO_4_)_2_ + CRS-38. Soil for the microcosm experiment was collected from the Research Farm of ICAR-IISS, Mau. Characteristics of the experimental soil were: pH 7.7, OC.37%, available N 313.85 kg ha^–1^, available P 79.36 kg ha^–1^, available K 599.64 kg ha^–1^, and DTPA-Zn 6.6 mg kg^–1^. The soil was mixed with Zn_3_(PO_4_)_2_ (2g kg^–1^) as Zn source, and each pot contained 3 kg of experimental soil.

Initially, four bioprimed seeds were sown in each pot, and, after germination, two plants per pot were maintained each throughout the experimentation. No additional fertilizers were applied during the experimentation. Moisture in the pots was maintained at field capacity by adding sterile distilled water as and when needed. The experiment was carried out in two sets. In the first set, five replicates of wheat plants were harvested after 30 days and utilized for recording the observations. However, the remaining five replicates were maintained up to the harvest stage, and data were recorded at maturity.

##### Analysis of Plant Samples

After 30 days of sowing, plants were harvested from the first set of experiments and data were recorded. Shoot length, root length, and dry weight of roots and shoots were recorded using standard agronomic procedures. For estimation of Zn content, plant samples (roots and shoots) were dried, ground to fine powder, and digested in a di-acid mixture containing nitric acid and perchloric acid (5:4 v/v) at 320*^o^*C for 1 h, and Zn content (μg Zn g^–1^ plant material) was measured using an atomic absorption spectrophotometer. For further analyses, plants were sampled from the second set of experiments after 120 days of sowing and brought to the laboratory. Roots and shoots were separated and washed in running tap water. Thereafter, the total length and fresh and dry weight of the shoots and roots were recorded. Seed yield (g plant^–1^) was also recorded. Zinc content in the shoot, root, and grain samples was estimated using the above-described method.

##### Expression Analyses of ZIP Transporter Genes

Quantitative real time-PCR (qPCR) analysis was performed to elucidate the expression and accumulation of transcript of ZIP transporter genes in different parts of wheat after 30 days of sowing, as per the method described by Singh et al. (2021). From the first set of experiments, root, shoot, and leaf samples were collected from each treatment in triplicates, brought to the laboratory, and quick-frozen in liquid nitrogen to isolate total RNA. RNA extraction was performed using an RNA isolation kit (Agilent, Mumbai, India) following the instructions of the manufacturer. cDNA synthesis was performed using a cDNA synthesis kit (BioRAD, Gurugram, Haryana, India) following the protocols of the manufacturer. The quality and quantity of cDNA were analyzed using Nanodrop 2000c (Thermo Fisher Scientific, Waltham, MA, United States). The housekeeping genes *actin* and *SuccDH* were used as an endogenous standard to normalize the quantitative expression data of *TaZIP1, TaZIP3, TaZIP5, TaZIP6, TaZIP7, TaZIP10, TaZIP13*, and *TaZIP15* genes in wheat. Gene expression was analyzed using gene-specific primers (Supplementary Table 2). A BioRAD Real-Time PCR system (MJ MiniOpticon System; BioRAD) was used for expression analyses, and relative transcription levels were calculated using the 2^–ΔΔ*CT*^ method (Livak and Schmittgen, 2001).

### Statistical Analysis

The glasshouse experiments were repeated twice for two consecutive years (2019 and 2020), and a pool analysis was performed. To test the significance of zinc-solubilizing rhizobacterial strains, a one-way analysis of variance was performed using Monitab 170. Means were separated by least significant differences (LSDs) at 95% significance level (*p* ≤ 0.05). A principal component analysis of strains based on variables was performed using SPSS version 16.0. Data were compared by Duncan’s multiple range test (DMRT) at *p* ≤ 0.05. Statistical software Origin version 9.0 and Microsoft Excel (Window 10) were used to prepare graphs.

## Results

### Screening of Zinc-Solubilizing Rhizobacteria

The zinc solubilization efficacy of rhizobacterial isolates was determined by measuring the diameter of halo zones formed in a growth medium supplemented with three different zinc compounds, viz. zinc oxide, zinc phosphate, and zinc carbonate. Among 15 rhizobacterial isolates, 11, viz., CRS-9, CRS-17, CRS-30, CRS-38, CRS-42, CRS-43, CRS-45, CRS-47, CRS-55, CRS-57, and CRS-77 showed varying degrees of zinc solubilization (zone ranging from 12.33 to 25.67 mm) (Table 1). However, all the fifteen rhizobacterial isolates did not solubilize zinc oxide and zinc carbonate. Out of the 11 zinc-solubilizing bacteria, four isolates, i.e., CRS-9 (21.33 mm), CRS-17 (25.67 mm), CRS-30 (24.33 mm), and CRS-38 (23.67 mm) were selected as potential zinc solubilizing rhizobacteria, and these isolates were chosen for further study (Supplementary Figure 1). Based on ZSI and ZSE, the most efficient bacterial isolates were CRS-17, CRS-38, CRS-30, and CRS-9 (Table 1). Furthermore, the rhizobacterial strains, viz., CRS-17, CRS-30, CRS-38, and CRS-9 showed maximum zinc phosphate solubilization efficiency (427.79, 317.39, 253.57, and 237.04%, respectively) (Table 1). The isolates, namely, CRS-17, CRS-30, CRS-9, and CRS-38 produced net soluble zinc a 6.1, 5.9, 5.63, and 5.6 μg ml^–1^, respectively, in zinc phosphate, and in broth, with corresponding pH decline to 4.5, 5.3, 5.2, and 4.8, respectively, from pH 6.7 (Table 1). Considering both qualitative and quantitative assays, the study indicated that all the four isolates are efficient zinc solubilizers. In general, the pH declined because of inoculation from 6.7 to 4.5 in zinc phosphate as against the absolute control. Maximum release of soluble zinc occurred in the liquid medium supplemented with zinc phosphate at the lowest mean pH value of 4.5, suggesting that solubilization is dependent on reduction of pH either by organic acid production or proton extrusion by bacteria (Table 1). Furthermore, the production of gluconic acid as one of the organic acids was measured with the assumption that it was a predominant organic acid that plays a crucial role in zinc solubilization.

**TABLE 1:** Efficacy of rhizobacterial isolates in insoluble solubilize zinc compounds under laboratory conditions.

Isolates	Zinc oxide	Zinc carbonate	Zinc phosphate *	Halo zone (mm)	ZSI	ZSE (%)	Zinc concentration (μg ml)	pH
CRS-9	–	–	++++	21.33 ± 1.05	11.37 ± 0.35	237.037	5.63 ± 0.21	5.2
CRS-17	–	–	++++	25.67 ± 2.15	10.28 ± 0.75	427.778	6.10 ± 0.14	4.5
CRS-26	–	–	–	–	–	–	–	–
CRS-30	–	–	++++	24.33 ± 1.33	10.84 ± 0.42	317.391	5.90 ± 0.26	5.3
CRS-37	–	–	–	–	–	–	–	–
CRS-38	–	–	++++	23.67 ± 1.65	11.87 ± 0.76	253.571	5.60 ± 0.37	4.7
CRS-42	–	–	+++	16.33 ± 1.01	10.04 ± 0.86	204.13	4.27 ± 0.45	4.9
CRS-43	–	–	+++	20.00 ± 0.95	10.98 ± 0.56	230.68	4.57 ± 0.25	4.8
CRS-45	–	–	+++	13.33 ± 0.45	9.67 ± 0.67	166.63	4.07 ± 0.33	4.7
CRS-47	–	–	+++	13.33 ± 0.66	8.22 ± 0.35	222.17	4.17 ± 0.30	5.1
CRS-50	–	–	–	–	–	–	–	
CRS-54	–	–	–	–	–	–	–	–
CRS-55	–	–	+++	14.67 ± 0.25	10.09 ± 0.27	176.11	4.37 ± 0.20	5.0
CRS-57	–	–	+++	12.33 ± 0.48	9.81 ± 0.63	148.02	3.73 ± 0.15	4.7
CRS-77	–	–	+++	17.67 ± 0.67	10.45 ± 0.25	212.12	4.60 ± 0.41	5.0

*+++, moderated activity; ++++, strong activity, -, no activity; ZSI, zinc solubilization index; ZSE, zinc solubilization efficiency; data are mean ± standard deviation (n = 5).

### Production of Gluconic Acid by Zinc Solubilizing Rhizobacteria

**FIGURE 1:**
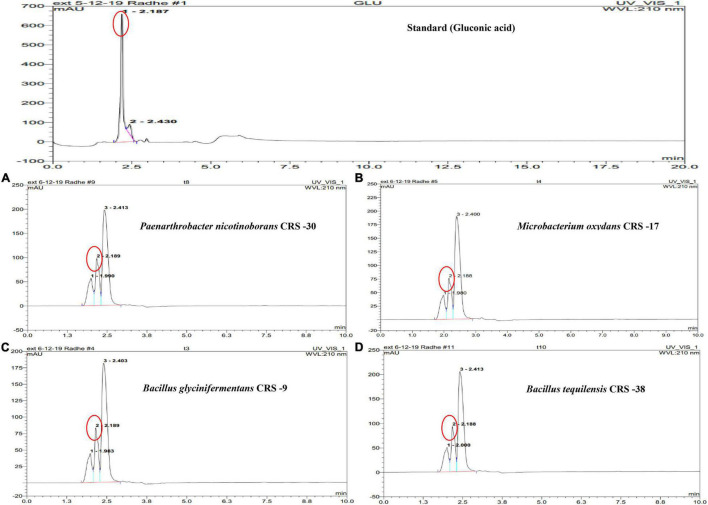
High-performance liquid chromatography (HPLC) chromatogram depicting the peaks of gluconic acid secreted by zinc-solubilizing rhizobacteria. Chromatograph of gluconic acid as standard (top) for a comparative study on its retention time (2.18 min) with the selected strains. Chromatographs of the samples of strains **(A)** CRS-30, **(B)** CRS-17, **(C)** CRS-9, and **(D)** CRS-38 of the 10-day cultured supernatant. Peak identified as gluconic acid is indicated through circle.

Production of gluconic acid in the presence of zinc phosphate by the bacterial isolates used in the study was determined by HPLC. The retention time for the gluconic acid standard was 2.187 min (Figure 1). It was observed that all the isolates produced gluconic acid in the medium supplemented with zinc phosphate, as depicted by peak and retention times. Peak height and area of gluconic acid in the chromatograph of *P. nicotinovorans* CRS-30 were found to be more compared to the other isolates. HPLC results revealed that a significantly higher amount of gluconic acid was produced by CRS-30 (189.41 μg ml^–1^) followed by CRS-38 (174.66 μg ml^–1^) and CRS-9 (153.29). However, the least amount of gluconic acid (144.4 μg ml^–1^) was recorded for CRS-17.

### Plant Growth-Promoting Traits

The selected zinc-solubilizing rhizobacterial isolates were further characterized for PGP traits, *viz.*, P solubilization, K solubilization, IAA production, ACC deaminase activity, siderophore production, and ammonia production (Supplementary Table 3). All the four isolates, CRS-9, CRS-17, CRS-30, and CRS-38, exhibited strong PGP traits and solubilize P and K except CRS-17, which lacks potassium solubilization ability. Similarly, all the isolates were found to produce varying levels of IAA in culture filtrate wherein CRS-30 showed highest IAA production (17.16 μg ml^–1^) followed by CRS-38 (8.61 μg ml^–1^), CRS-9 (6.16 μg ml^–1^), and CRS-17 (6.43 μg ml^–1^). Except for CRS-38, the other three isolates were found to be negative for ACC deaminase activity. All the bacterial isolates produced siderophore except CRS-9, and only two isolates, viz., CRS-9 and CRS-38, produced ammonia in the respective media (Supplementary Table 3).

### Biochemical and Molecular Characterization of Isolates

Four rhizobacterial isolates, viz., CRS-9, CRS-17, CRS-30, and CRS-38, were characterized on a cultural basis as per *Bergey’s Manual of Determinative Bacteriology* (Supplementary Table 4). All the strains tested showed different colony morphology, were Gram-positive, and rod-shaped, and two of them (CRS-9 and CRS-17) formed endospores. All the strains showed different reactions for catalase, oxidase, nitrate, H_2_S, citrate utilization, Voges Proskauer’s, esculin hydrolysis, methyl red, indol, ONPG, lysine, etc. (Supplementary Table 4).

**FIGURE 2:**
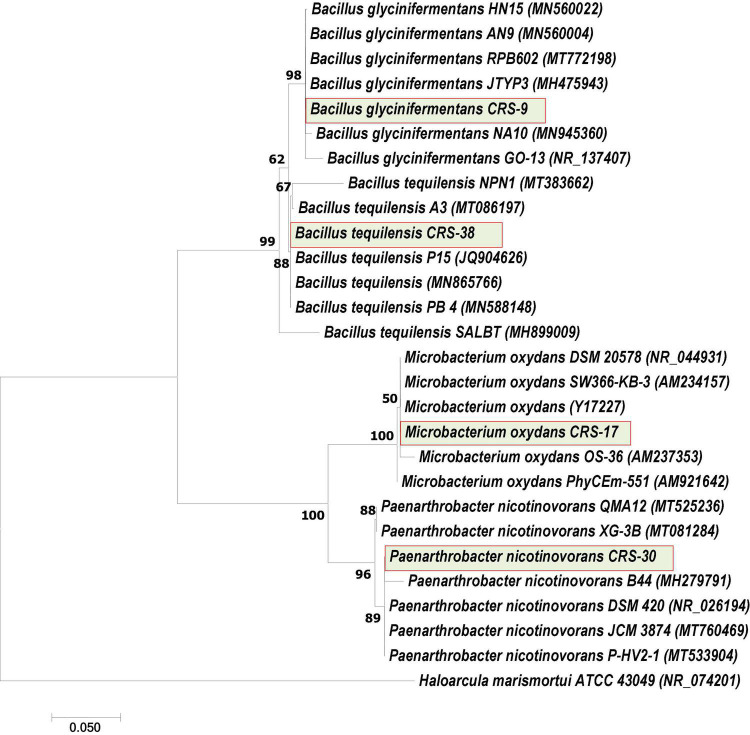
Neighbor-joining tree derived with CLUSTAL W and MEGA 5.0 by analysis of 16S rRNA gene sequences. The numbers at nodes indicate bootstrap support values, as calculated with MEGA 5.0. Bacterial strains used in this study are presented in highlighted box.

Based on 16S rRNA gene sequencing, these isolates were identified as *Bacillus glycinifermentans* CRS-9 (MH497203; 1,412 bp, 99.5% similarity), *Microbacterium oxydans* CRS-17 (MH497204; 1,368 bp, 100% similarity), *Paenarthrobacter nicotinovorans* CRS-30 (MH497212; 1,378 bp, 99.1% similarity), and *Bacillus tequilensis* CRS-38 (MH497217; 1,407 bp, 99.7% similarity) (Figure 2). Percent similarity, E-score, query coverage, etc., are presented in Supplementary Table 5. All the cultures were deposited in the National Agriculturally Important Microbial Culture Collection (NAIMCC), a microbial resource center of ICAR-NBAIM, Kushmaur, India (Supplementary Table 5).

### Root Colonization and Biofilm Formation

**FIGURE 3:**
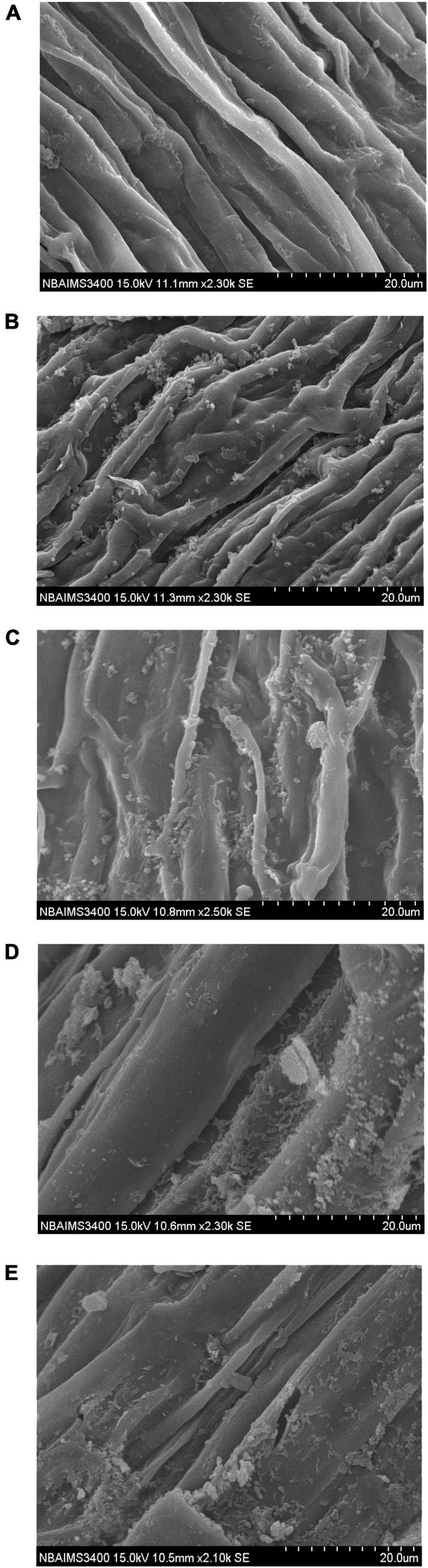
Scanning electron microphotographs showing root colonization and biofilm formation by selected strains under glasshouse conditions, **(A)** Absolute control, **(B)** *Bacillus glycinifermentans* CRS-9, and **(C)** *Microbacterium oxydans* CRS-17, **(D)** *Paenarthrobacter nicotinovorans* CRS-30, and **(E)** *Bacillus tequilensis* CRS-38.

Upon inoculation, the bacterial strains start colonizing radicals and grow along with the primary and secondary roots. Scanning electron microphotographs clearly indicated that the selected strains have a potential to colonize wheat roots. All the strains were found to colonize and formed biofilm in wheat roots. Results indicated that bacterial strains produced an ample amount of extracellular polysaccharides (EPSs) which facilitate the initial attachment and biofilm cells that are embedded in the EPS matrix along with root surface (Figure 3).

### Effects of Inoculation on Seed Germination and Vigor Indices

The results revealed that maximum seed germination (in%) was recorded in seeds treated with CRS-38 (89.10%) followed by CRS-30 (86.25%). However, least germination was recorded for the absolute control (76.29%) after 30 days of sowing (Table 2). Similarly, a significant increment in vigor index I was recorded in plants inoculated with CRS-38 (4,868.26) followed by CRS-30 (4,249.25), and CRS-17 (4,075.14) compared to the other treatments and absolute control (2,237.84). More or less similar trends were recorded in the case of vigor index-II (Table 2 and Supplementary Figure 2).

**TABLE 2:** Effect of seed inoculation on germination and vigor indices of wheat grown in pots under glasshouse conditions after 30 days of sowing.

Treatments	Germination (%)	Vigor index-I	Vigor index-II
Absolute control	76.29 ± 1.20*^d^*	2,237.84 ± 6.50*^f^*	50.56 ± 1.96*^f^*
Zn_3_(PO4)_2_	77.05 ± 1.33*^d^*	2,568.33 ± 8.25*^e^*	56.67 ± 2.50*^e^*
Zn_3_(PO4)_2_ + CRS-9	82.76 ± 1.25*^c^*	3,565.58 ± 10.02*^d^*	105.96 ± 2.33*^c^*
Zn_3_(PO4)_2_ + CRS-17	82.05 ± 1.66*^c^*	4,075.15 ± 11.25*^c^*	113.39 ± 1.75*^b^*
Zn_3_(PO4)_2_ + CRS-30	86.25 ± 1.50*^b^*	4,249.25 ± 9.66*^b^*	90.56 ± 2.25*^d^*
Zn_3_(PO4)_2_ + CRS-38	88.10 ± 1.96*^a^*	4,868.99 ± 8.50*^a^*	137.44 ± 3.36*^a^*

*Data are mean ± standard deviation (n = 5), and values within a column followed by a different letter are significantly different at p < 0.05.*

### Plant Experiments

Of the three zinc sources tested, only zinc phosphate was solubilized by the selected rhizobacterial isolates. Hence, zinc phosphate was selected as a source of zinc in plant experiments under microcosm conditions. Based on zinc phosphate solubilization and multiple PGP traits, the four potential rhizobacterial strains were selected for plant experiments. In these experiments, each treatment was replicated ten times.

#### After 30 Days of Plant Growth

##### Plant Growth Attributes

**FIGURE 4:**
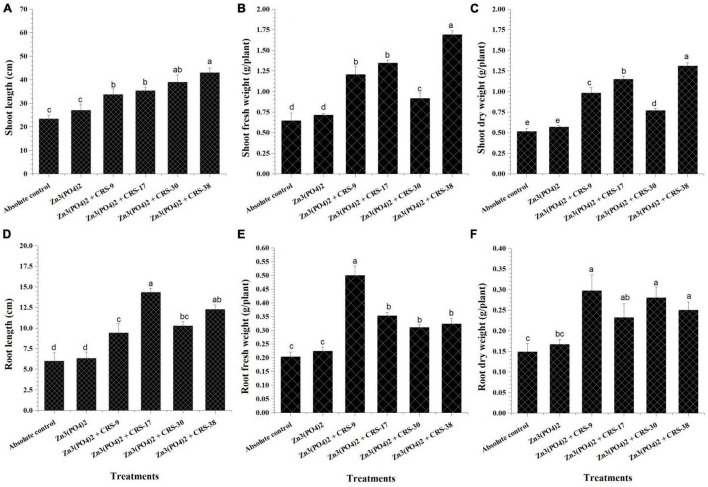
Effect of zinc solubilizing rhizobacteria on growth parameters of plants harvested after 30 days (first set). **(A)** Shoot length, **(B)** Fresh weight of shoots **(C)** Dry weight of shoots, **(D)** Root length **(E)** Fresh weight of roots and **(F)** Dry weight of roots in comparison with absolute control. Data are mean (*n* = 5) and vertical bar represents standard deviation. Data with different letters show significant difference in column data in randomized block design test at *p* < 0.05 under Duncan’s multiple range test.

The first set of plant experiments showed that all the strains considerably increased the parameters of root and shoots as compared to plants from absolute control (Figures 4A–F). Maximum shoot length was recorded for *B. tequilensis* CRS-38-inoculated plants; *B. glycinifermentans* CRS-9, *P. nicotinovorans* CRS-30, and *M. oxydans* CRS-17 showed minor differences (Figure 4A). Inoculation with *B. tequilensis* CRS-38 significantly increased the fresh and dry weight of shoots compared to that with absolute control and control + Zn_3_ (PO_4_)_2_. All the other isolates significantly increased the fresh and dry weight of shoots of the inoculated plants compared to the absolute control, but this increase was not significant when compared to control + Zn_3_ (PO_4_)_2_ (Figures 4B,C). Seed treatment with *B. glycinifermentans* CRS-9 and *P. nicotinovorans* CRS-30 resulted in a substantial increase in root length. However, *M. oxydans* CRS-17 and *B. tequilensis* CRS-38 seem to increase the root length of the plants significantly compared to the absolute control and control + Zn_3_ (PO_4_)_2_. Maximum root length was observed in plants that emerged from the seeds treated with CRS-17 followed by those treated with CRS-38, and minimum root length was recorded for plants that emerged from the seeds treated with CRS-9 compared to other inoculants (Figure 4D). Inoculation with *B. glycinifermentans* CRS-9 and *M. oxydans* CRS-17 significantly increased the fresh and dry root weight of the plants compared to that with the absolute control and control + Zn_3_ (PO_4_)_2_. A non-significant increase was observed in the fresh and dry root weight of plants inoculated with *P. nicotinovorans* CRS-30 and *B. tequilensis* CRS-38 compared to the absolute control and control + Zn_3_ (PO_4_)_2_ (Figures 4E,F).

##### Zinc Content

**FIGURE 5:**
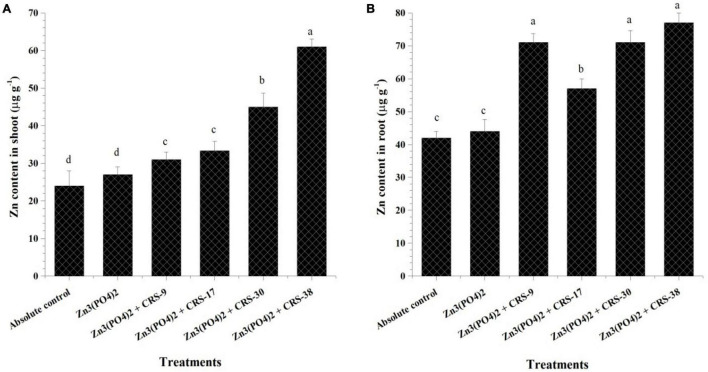
Estimation of zinc content in **(A)** shoots and **(B)** roots of inoculated and un-inoculated plants harvested after 30 days. Data are mean (*n* = 5), and vertical bar represents standard deviation. Data with different letters show significant difference in column data in the randomized block design test at *p* < 0.05 under Duncan’s multiple range test.

Inoculation with different zinc-solubilizing rhizobacterial strains significantly increased the zinc content in shoots and roots of the inoculated plants as compared to the absolute control plants (Figures 5A,B). Maximum zinc content was found in shoots of the plants inoculated with *B. tequilensis* CRS-38 (64.26 μg g^–1^) followed by those inoculated with *P. nicotinovorans* CRS-30 (45.25 μg g^–1^), *M. oxydans* CRS-17 (33.33 μg g^–1^), and *B. glycinifermentans* CRS-9 (31.26 μg g^–1^). However, least zinc content was recorded in shoots of the control plants (24.1 μg g^–1^) after 30 days of sowing (Figure 5A).

The zinc content of the roots was significantly higher than that of the shoots across the treatments (Figure 5B). The highest zinc content in the roots was recorded in plants treated with *Bacillus tequilensis* CRS-38 (77.50 μg g^–1^) followed by those treated with *B. glycinifermentans* CRS-9 (71.46 μg g^–1^), *P. nicotinovorans* CRS-30 (71.25 μg g^–1^), and *M. oxydans* CRS-17 (57.1 μg g^–1^). Least zinc content was found in roots of the absolute control plants (42.5 μg g^–1^) and plants supplemented with Zn_3_ (PO_4_)_2_ (44.25 μg g^–1^) after 30 days of sowing (Figure 5B).

##### Expression of ZIP Transporter Genes

**FIGURE 6:**
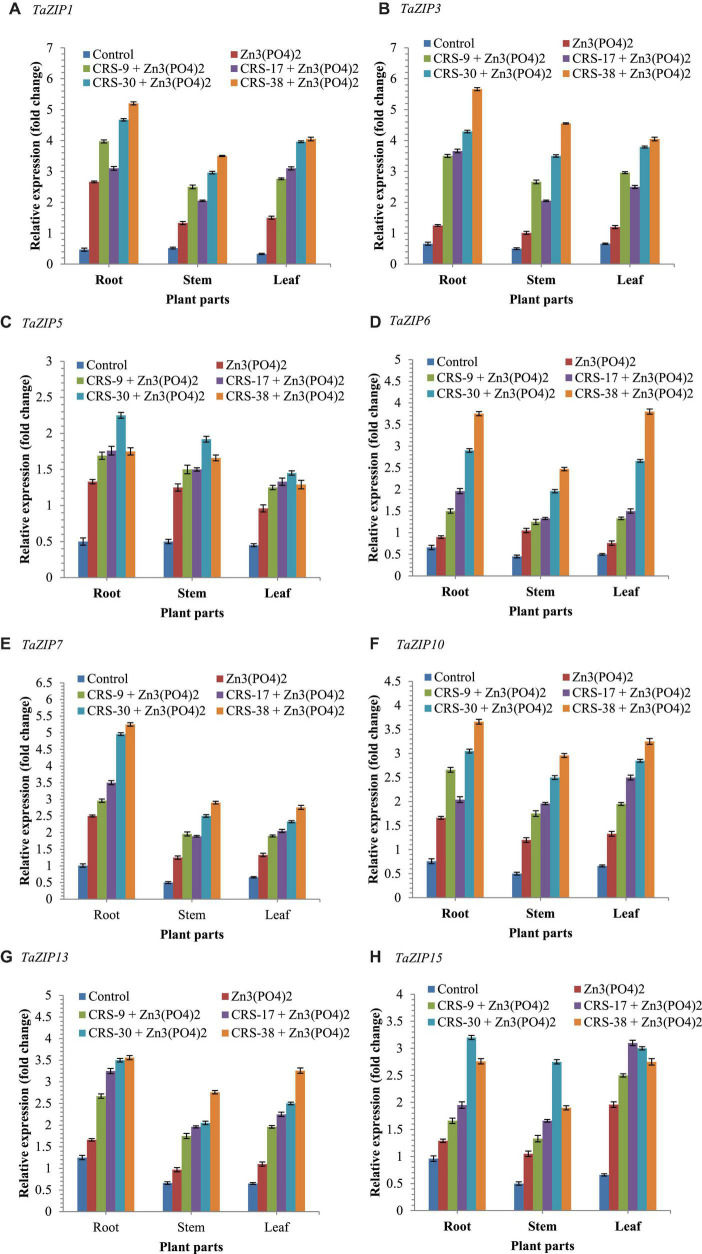
Effects of seed bioinoculation on expression profile of TaZIP transporter genes (fold change) in the wheat root, shoot and leaves grown at 30 days of sowing. **(A)** Relative expression (fold change) of *TaZIP1*, **(B)** Relative expression (fold change) of *TaZIP3*, **(C)** Relative expression (fold change) of *TaZIP5*, **(D)** Relative expression (fold change) of *TaZIP6*, **(E)** Relative expression (fold change) of *TaZIP7*, **(F)** Relative expression (fold change) of *TaZIP10*, **(G)** Relative expression (fold change) of *TaZIP13*, and **(H)** Relative expression (fold change) of *TaZIP15*.

In this investigation, 9 *TaZIP* transporters genes were taken for an expression study, and out of the 9 *TaZIP* transporters, 8 *TaZIP* transporter genes (*TaZIP1, TaZIP3, TaZIP5, TaZIP6, TaZIP7, TaZIP10, TaZIP13*, and *TaZIP15*) were found to be expressed in different parts of the plants after 30 days of sowing. The results revealed that significantly higher expression of all the 8 *TaZIP* transporters was recorded in the bacteria-inoculated plants supplemented with Zn_3_ (PO_4_)_2_ compared to the Zn_3_ (PO_4_)_2_ and absolute control plants (Figure 6). Interestingly, the expression of all the 8 *TaZIP* transporters genes was significantly higher in the roots than in shoots and leaves of the same plants across the treatments. In general, the expression of the 8 *TaZIP* transporters genes was higher in leaves than in stems but lower in roots of the same plants. Furthermore, it was observed that plants treated with bacterial inoculant CRS-38 + Zn_3_(PO_4_)_2_ showed maximum transcript level followed by CRS-30 + Zn_3_ (PO_4_)_2_. However, least expression was recorded in untreated control plants followed by plants amended with Zn_3_ (PO_4_)_2_ alone (Figure 6).

#### In Harvest Stage (120 Days of Sowing)

##### Plant Growth Attributes

**FIGURE 7:**
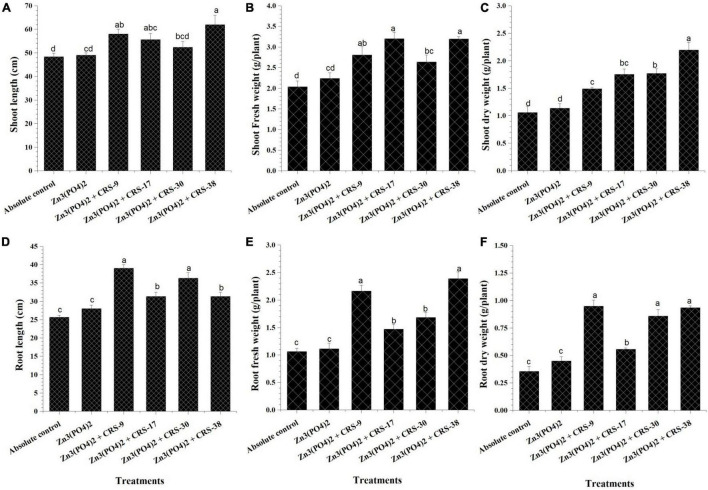
Effect of zinc-solubilizing rhizobacterial inoculation on growth parameters of plants harvested after 120 days (second set). **(A)** Shoot length, **(B)** fresh weight of shoots, **(C)** dry weight of shoots, **(D)** root length, **(E)** fresh weight of roots, **(F)** dry weight of roots in comparison with absolute control. Data are mean (*n* = 5), and the vertical bar represents standard deviation. Data with different letters show a significant difference in column data in the randomized block design test at *p* < 0.05 under Duncan’s multiple range test.

Harvesting of the wheat crop was done after 120 days of sowing, and data for shoot and root length, fresh and dry weight, Zn content in roots, shoots, and grains, and yield plant^–1^ were recorded (Figure 7). Significantly higher shoot length was recorded for plants inoculated with *B. tequilensis* CRS-38 + Zn_3_ (PO_4_)_2_ (62.5 cm) followed by *B. glycinifermentans* CRS-9 + Zn_3_ (PO_4_)_2_ (58.25 cm), and *M. oxydans* CRS-17 + Zn_3_ (PO_4_)_2_ (55.62 cm). Least shoot length was recorded for the absolute control (48.33 cm) and plants supplemented with Zn_3_(PO_4_)_2_ alone (49.1 cm) after 120 days of sowing (Figure 7A). Similarly, all the bacterial isolates showed a significant increase in root length of the plants whereas *B. glycinifermentans* CRS-9 + Zn_3_ (PO_4_)_2_ showed maximum increment in root length (39.25 cm) followed by *P. nicotinovorans* CRS-30 + Zn_3_ (PO_4_)_2_ (36.25 cm), *B. tequilensis* CRS-38 + Zn_3_ (PO_4_)_2_ (31.35 cm), and *M. oxydans* CRS-17 + Zn_3_ (PO_4_)_2_ (31.33 cm) when compared to the root length of the absolute control plants (25.66 cm) after 120 days of sowing (Figure 7D).

Similar trends were recorded for shoot and root fresh weight. Significantly higher fresh biomass of shoots and roots was recorded in plants inoculated with *B. tequilensis* CRS-38 + Zn_3_(PO_4_)_2_ (3.1 and 2.38 g plant^–1^, respectively) after 120 days of sowing (Figure 7B). Moreover, maximum dry weight accumulation in shoots was observed in plants inoculated with *B. tequilensis* CRS-38 + Zn_3_(PO_4_)_2_ (2.19 g plant^–1^). Least shoot dry weight was recorded for absolute control plants (1.05 g plant^–1^). However, maximum root dry weight was observed in *B. glycinifermentans* CRS-9 + Zn_3_(PO_4_)_2_ and *B. tequilensis* CRS-38 + Zn_3_(PO_4_)_2_ (0.94 and.93 g plant^–1^, respectively) compared to the other treatments after 120 days of sowing (Figures 7C,F).

##### Zinc Content

**FIGURE 8:**
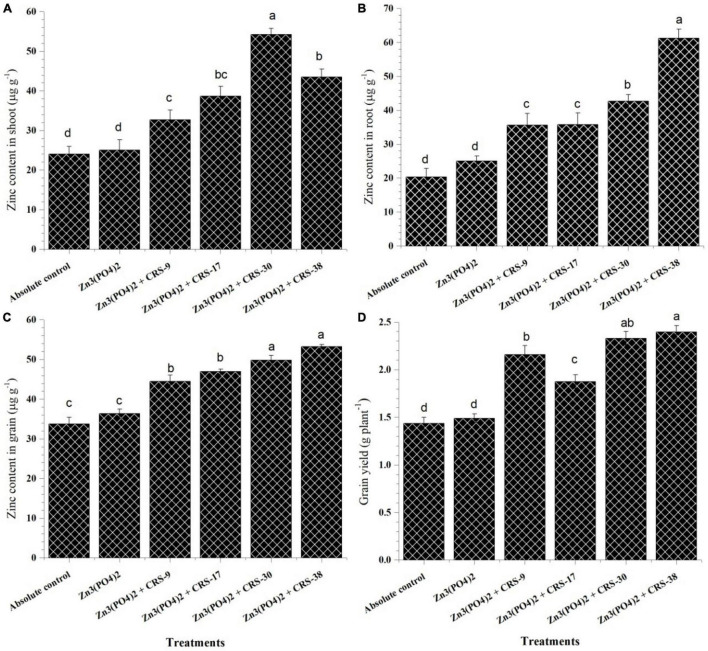
Estimation of zinc content in **(A)** shoots, **(B)** roots, **(C)** grains, and **(D)** grain yield of inoculated and un-inoculated plants harvested after 120 days (second set). Data are mean (*n=5*) and vertical bar represents standard deviation. Data with different letters show significant difference in column data in randomized block design test at *p* < 0:05 under Duncan’s multiple range test.

The zinc content of shoots, roots, and grains was recorded in plants inoculated with different zinc-solubilizing rhizobacterial isolates after 120 days of sowing (Figure 8). Significantly higher Zn content in shoots was recorded in plants inoculated with *P. nicotinovorans* CRS-30 + Zn_3_ (PO_4_)_2_ (54.25 μg g^–1^) followed by *B. tequilensis* CRS-38 + Zn_3_ (PO_4_)_2_ (43.5 μg g^–1^), and *M. oxydans* CRS-17 + Zn_3_ (PO_4_)_2_ (38.62 μg g^–1^) (Figure 8A). However, maximum Zn content in roots was observed in plants inoculated with *B. tequilensis* CRS-38 + Zn_3_ (PO_4_)_2_ (61.25 μg g^–1^), while it was least with the absolute control (20.33 μg g^–1^) after 120 days of sowing (Figure 8B). In general, plant roots have higher Zn content than shoots across the treatments including the absolute control.

Similar to Zn content in the shoot, significantly higher Zn content in grain was observed in the plant inoculated with *B. tequilensis* CRS-38 + Zn_3_ (PO_4_)_2_ (53.25 μg g^–1^) followed by *P. nicotinovorans* CRS-30 + Zn_3_ (PO_4_)_2_ (49.82 μg g^–1^), *M. oxydans* CRS-17 + Zn_3_ (PO_4_)_2_ (46.98 μg g^–1^) and *B. glycinifermentans* CRS-9 + Zn_3_ (PO_4_)_2_ (44.50 μg g^–1^) as against the absolute control (33.76 μg g^–1^) and the plant supplemented with Zn_3_ (PO_4_)_2_ alone (36.35 μg g^–1^) (Figure 8C). The significant increase in zinc content of grains of the inoculated plants showed the importance of bacterial inoculants across the treatments. These results indicate the efficacy of the selected zinc solubilizing-rhizobacteria in enhancing the bioavailability of zinc and mobilizing it toward wheat grains. Inoculation with *B. glycinifermentans* CRS-9, *M. oxydans* CRS-17, *P. nicotinovorans* CRS-30, and *B. tequilensis* CRS-38 significantly increased grain yield compared to that with the absolute control and supplementation with Zn_3_ (PO_4_)_2_ alone. Maximum grain yield was recorded for plants inoculated with *B. tequilensis* CRS-38 + Zn_3_ (PO_4_)_2_ (2.39 g plant^–1^) followed by *P. nicotinovorans* CRS-30 + Zn_3_ (PO_4_)_2_ (2.33 g plant^–1^) compared to the other treatments (Figure 8D).

**FIGURE 9:**
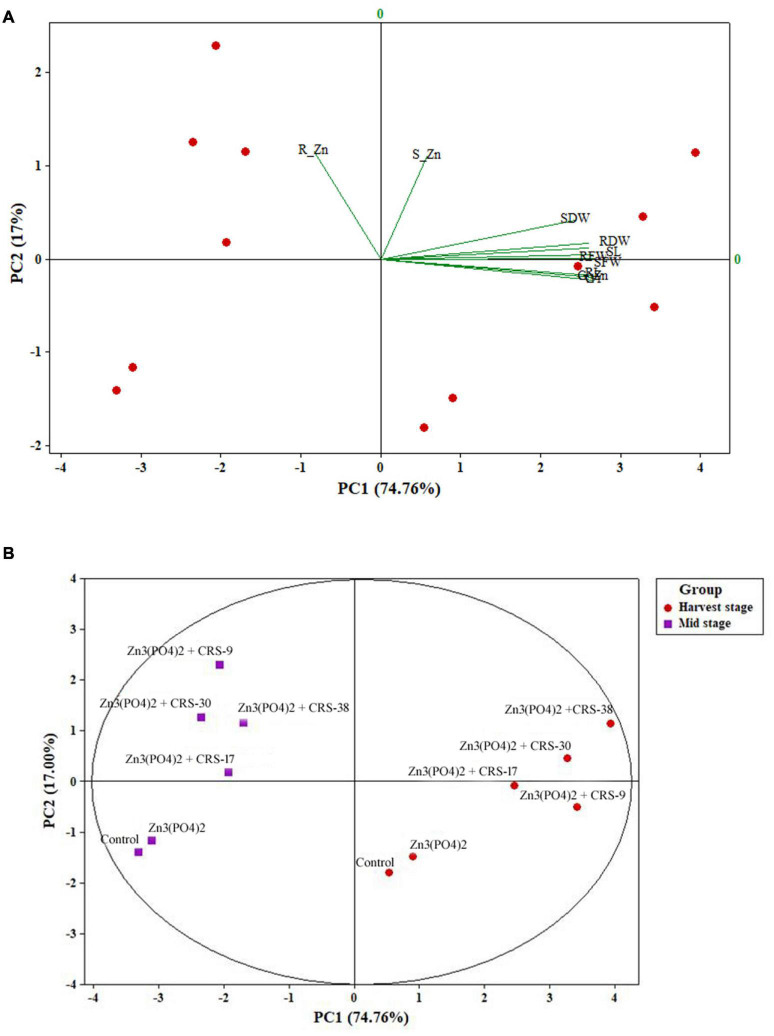
**(A)** Grouping of variables of wheat crops based on principal component scores (PC1 and PC2) and **(B)** grouping of rhizobacterial strains based on principal component scores (PC1 and PC2) derived from variables (SWD, RWD, RL, SL, RFW, R_Zn, S_Zn, and G_Zn) of wheat crop after 30 and 120 days (harvest stage) of wheat crop sowing.

The position of different plant growth attributes, such as fresh and dry weight of root and shoot, and Zn content as influenced by rhizobacterial strains in the four zones of biplot of PCA are depicted in Figure 9. The PCA comprising two principal components (PC1 74.76% and PC2 17%) accounted for 91.76% of the variance (Figure 9A). The interpretation among the different plant growth parameters and Zn content was more evident through the projection of PC1 and PC2. All plant growth attributes occupied a position solely in the right upper part of the biplots and showed a positive correlation among them. However, they were negatively correlated with root zinc content. Furthermore, it was observed that rhizobacterial strains + Zn_3_ (PO_4_)_2_ formed a separate group, and that Zn_3_ (PO_4_)_2_ alone and the absolute control formed separate groups in both mid stage (30 DAS) and harvest stage. However, two separate clusters formed during the mid-stage and harvest stage. PC1 and PC2 indicated that CRS-38 and CRS-30 showed a highly significant correlation compared to the other two rhizobacterial strains in the mid stage with respect to PC2. In contrast, CRS-38 and CRS-30 showed higher significance than the other two rhizobacterial strains in the harvest stage with respect to PC1 (Figure 9B). It is evident that the rhizobacterial strains did not express their potential completely in the mid stage, and the full potential of the bacterial strains was recorded only in the harvest stage. However, factors responsible for this differential effect of rhizobacteria on wheat crops need to be identified.

## Discussion

Zinc is an essential micronutrient required for the overall growth and development of a plant. Zinc deficiency is prevalent globally in crops, leading to considerable economic losses. Alleviation of zinc deficiency through the application of zinc fertilizers may not be environmentally friendly and cost-effective. Many strategies are available that help in enhancing zinc levels in plants without harming the environment (Cakmak, 2008; White and Broadley, 2011). One such strategy is harnessing the potential of rhizobacteria that can mobilize unavailable zinc and increase assimilation of zinc, and accelerate growth and yield of plants. Rhizobacteria play an important role in environmental geo-cycling processes, such as solubilization of immobilized metal ion forms, which can be suitably taken up by plants. Zinc-solubilizing rhizobacteria can be used as an alternative strategy for enhancing zinc uptake by plants because of their ability to solubilize Zn through secretion of organic acids, protein extrusion, and production of chelating agents (Nahas, 1996; Seshadri et al., 2002). Thus, nowadays, scientific interest has increased in utilization of zinc-solubilizing rhizobacteria because of their role in increasing zinc uptake in plants. Therefore, the main focus of this study was to characterize potential zinc-solubilizing rhizobacterial isolates and their application for overall plant growth, with special reference to zinc biofortification in wheat.

In this study, different rhizobacteria were isolated from chickpea rhizospheric soils of the Indo-Gangetic plains of Northern India. These bacteria were evaluated for their ability to solubilize zinc using different zinc compounds. Out of 15 rhizobacterial isolates, only 11 showed zinc-solubilizing potential in a medium supplemented with zinc phosphate Zn_3_ (PO_4_). The variation in the diameter of halo zone formation indicated a variation in the degree of solubilization. Various studies have reported variation in solubilization by bacterial strains (Sharma et al., 2011; Ramesh et al., 2014a; Khande et al., 2017). In this investigation, the isolates showed varying levels of ZSI and ZSE ranging from 8.22 to 11.87 and 148 to 427%, respectively. These findings are corroborated by the study of Ramesh et al. (2014b), wherein they found ZSE of up to 175% by strains of *Bacillus aryabhattai*. In this study, it was found that different rhizobacterial strains produced a varying level of zinc content in a liquid medium that ranged from 2.47–6.1 μg/ml. The results obtained in this study are consistent with the findings of Sharma et al. (2011), wherein they showed that the zinc concentration of different *Bacillus* spp. ranged between 2.23 and 4.87 μg/ml. This study showed that zinc-solubilizing rhizobacterial isolates were able to decrease pH from 7 to 4.5. The reduction in pH of the liquid media suggested that these rhizobacterial isolates produce organic acids. This finding is in consonance with reports by other workers (Saravanan et al., 2004) who reported a reduction in the pH of liquid media by rhizobacteria owing to their ability to produce different organic acids. Similar observations have been reported by several other workers (Desai et al., 2012; Kumari et al., 2016) who reported that gluconic acid is the main organic acid besides other organic acids in solubilization of insoluble minerals.

Promising zinc-solubilizing bacterial isolates, viz., CRS-9, CRS-17, CRS-30, and CRS-38 were identified as *B. glycinifermentans*, *M. oxydans*, *P. nicotinovorans*, and *B. tequilensis*, respectively. Scanning electron microphotographs clearly showed that all the four strains tested colonized wheat roots and developed biofilm in the root matrix. These bacterial genera have also been reported to colonize plant rhizosphere and increase plant growth (Çakmakçı et al., 2017; Singh et al., 2018a). Various species of *Bacillus* have been extensively studied for their potential plant growth-promoting activity (Mumtaz et al., 2017; Dinesh et al., 2018). In this study, *B. tequilensis* CRS-38 and *B. glycinifermentans* CRS-9 have been found to possess significant zinc solubilization activity and PGP traits. However, to the best of our knowledge, this is the first report on zinc solubilization and PGPR activity by *B. glycinifermentans*, although the selected rhizobacteria have been previously isolated from Ohio soil and soybean food in Korea (Zeigler, 2016). Similarly, species belonging to *Microbacterium* have also been found to exhibit multiple plant growth-promoting characteristics (Ouertani et al., 2020). In this study, *M. oxydans* CRS-17 has shown numerous plant growth-promoting activities (Supplementary Table 3) and has also been found to solubilize zinc in its insoluble form (Table 1). Reports have shown that the plant growth-promoting strain of *M. oxydans* increases the overall biomass of rape plants grown on metal-contaminated soil (Ren et al., 2019). However, reports on zinc solubilization by *M. oxydans* are lacking elsewhere. *Paenarthrobacter* spp. has been reported to produce siderophores and indole acetic acid, and fix nitrogen. *P. nicotinovorans* (formerly known as *Arthrobacter nicotinovorans*) was previously known for its ability of P solubilization, IAA production, and ACC deaminase activity, and enhances P uptake and plant biomass in maize (Pereira and Castro, 2014). In this study, *P. nicotinovorans* CRS-30 also showed multiple PGPR traits along with solubilization of zinc, phosphorus, and potassium (Supplementary Table 3).

To the best of our knowledge, the three strains, *B. glycinifermentans* CRS-9, *M. oxydans* CRS-17, and *P. nicotinovorans* CRS-30 utilized in this study have not yet been reported for their zinc-solubilizing ability, zinc biofortification, and contribution in promoting growth of wheat plants. Inoculation of wheat plants with these strains resulted in a significant positive difference in root and shoot length, biomass, and zinc content compared to that with the absolute control. At 30 DAS, *B. glycinifermentans* CRS-9 and *P. nicotinovorans* CRS-30 were found to significantly increase zinc concentration in the shoots, while the maximum increase in zinc content in the roots was recorded for inoculation with *B. tequilensis* CRS-38 followed by that with *P. nicotinovorans* CRS-30 and *B. glycinifermentans* CRS-9. However, zinc concentration in the shoots and roots was maximally increased by *P. nicotinovorans* CRS-30 and *B. tequilensis* CRS-38 at the time of harvesting (120 DAS). *B. glycinifermentans* CRS-9, *P. nicotinovorans* CRS-30, and *B. tequilensis* CRS-38 were found to be promising when the zinc content of wheat grains was analyzed at the time of harvest (120 DAS). These observations support that these PGPR genera have significantly contributed to enhancement of the bioavailability of zinc in the shoots, roots, and grains of the wheat plant and provide it with more available zinc as against absolute control.

The ZIP (Zn-regulated, iron-regulated transporter-like protein) transporter family is one of the key gene families regulating the uptake, transport, and accumulation of Zn and Fe in plants (Singh et al., 2017, 2018a,b). It is a widely studied transporter playing an important role in several developmental processes, such as plant growth, uptake, and translocation of key microelements, tissues differentiation, biofortification, etc., in plant systems (Velmourougane et al., 2017). Although the ZIP protein family has been widely and perhaps systematically studied in *Arabidopsis*, a model plant, and many other plant species, the significance of this family in wheat upon inoculation of *B. glycinifermentans* CRS-9, *M. oxydans* CRS-17, and *P. nicotinovorans* CRS-30 is not well-understood at present. The results of glasshouse experiments with *B. glycinifermentans* CRS-9, *M. oxydans* CRS-17, and *P. nicotinovorans* CRS-30 clearly showed the significance of these inoculants on uptake, translocation, and bioaccumulation of Zn in wheat. The expression analysis clearly showed that the *TaZIPs* genes were significantly upregulated and highly expressed in the roots, and that nine *TaZIP* genes were up-regulated in the roots, stems, and leaves of plants bioprimed with selected microbial inoculants in the presence of zinc phosphate after 30 days of sowing.

Enhanced zinc content in the roots and shoots as against absolute plants is in line with the previous reports wherein inoculation of plants with PGPR led to enhanced yield, plant growth, and improved nutrition. Besides the increase in the overall yield of the plant, PGPR have been reported to be useful in countering nutrient deficiencies and have received attention for their use as biofertilizers. Ramesh et al. (2014b) reported increased mobilization of zinc by zinc-solubilizing *Bacillus aryabhattai* in wheat and soybean. Furthermore, the application of PGPR boosted the translocation of zinc toward wheat grains, and this is because of the ability of rhizobacteria to successfully execute plant-microbe interactions, viz., induction of physiological processes, mineralization, and solubilization (Lucas et al., 2014; Wang et al., 2014). Zinc solubilization is important in improving plant growth and cannot be overlooked. Zinc solubilization by PGPR is a comparatively new approach, but not many strains have yet been reported for this activity. This investigation explored the potential of *B. tequilensis* and *P. nicotinovorans* for their use as biofertilizers to overcome zinc deficiency in wheat crops.

## Conclusion

Our study on zinc-solubilizing, plant growth-promoting rhizobacteria in wheat revealed that their inoculation is an effective method for enhancing the growth of wheat and maintaining nutritional quality. Out of four rhizobacteria, *B. tequilensis* CRS-38 and *P. nicotinovorans* CRS-30 inoculation improved the expression of eight *TaZIP* transporter genes in the roots after 30 days of wheat growth and increased grain yield and grain with zinc in wheat crop. The zinc solubilization potential of *B. glycinifermentans* CRS-9, *M. oxydans* CRS-17, and *P. nicotinovorans* CRS-30 has been reported for the first time in this study. These bacteria could be used as bio-input for improving productivity while combating nutrient deficiency in wheat as an option both in conjunction with and without chemical fertilizers, and such practice would help in achieving the objectives of SDG1, SDG2 and SDG3 of the United Nations by 2030.

## Data Availability Statement

The datasets presented in this study can be found in online repositories. The names of the repository/repositories and accession number(s) can be found in the article/Supplementary Material.

## Author Contributions

SS, AV, PS, and AS conceptualized the idea for this research study and corrected base manuscript. RY designed the experiments, and conducted and developed the first draft of research manuscript. DM and US conducted the transporter gene expression experiments. MR and MK analyzed the data using different kinds of software and interpreted the data thereafter. All authors read and gave consent to the final draft of manuscript.

## Conflict of Interest

The authors declare that the research was conducted in the absence of any commercial or financial relationships that could be construed as a potential conflict of interest.

## Publisher’s Note

All claims expressed in this article are solely those of the authors and do not necessarily represent those of their affiliated organizations, or those of the publisher, the editors and the reviewers. Any product that may be evaluated in this article, or claim that may be made by its manufacturer, is not guaranteed or endorsed by the publisher.

## Footnotes

1
https://www.ncbi.nlm.nih.gov
