# Supplementary material for: Modulation in Biofertilization and Biofortification of Wheat Crop by Inoculation of Zinc-Solubilizing Rhizobacteria
Source: Front Plant Sci. 2022 Feb 25;13:777771. doi: 10.3389/fpls.2022.777771 (PMC8914200; doi:10.3389/fpls.2022.777771)
Supplement: Supplementary file 1 [file Data_Sheet_1.docx]

**Supplementary Materials**

**Supplementary Table 1.** Geographical locations from where zinc solubilizing rhizobacterial strains were isolated.

| **S.No.** | **Isolate** | **Locations** | **Coordinates** | **Year of isolation** |
| --- | --- | --- | --- | --- |
|  | CRS-9 | Kadhikueya, Payagpur, Baharaiech, Uttar Pradesh (India) | 27°24'0.3708''N 81°46'33.3804''E | **2016** |
|  | CRS-17 | Kurebhar, Sultanpur, Uttar Pradesh (India) | 26°24' 46.1772''N 82°07'17.4252''E | **2017** |
|  | CRS-26 | Akabarpur, Ambedkar Nagar, Uttar Pradesh (India) | 26°25'37.1532''N 82°31'59.2032''E | **2017** |
|  | CRS- 30 | Paratwal, Maharaj Ganj, Uttar Pradesh (India) | 26°58'5.7756''N 83°36'5.6916''E | **2018** |
|  | CRS- 37 | Paratwal, Maharaj Ganj, Uttar Pradesh (India) | 26°58'5.7756''N 83°36'5.6916''E | **2018** |
|  | CRS-38 | Paratwal, Maharaj Ganj, Uttar Pradesh (India) | 26°58'5.7756''N 83°36'5.6916''E | **2017** |
|  | CRS-42 | Chakia, Chandauli, Uttar Pradesh (India) | 25°2'57.1632''N 83°13'16.1544''E | **2016** |
|  | CRS-43 | Chakia, Chandauli, Uttar Pradesh (India) | 25°2'57.1632''N 83°13'16.1544''E | **2017** |
|  | CRS-45 | Chakia, Chandauli, Uttar Pradesh (India) | 25°2'57.1632''N 83°13'16.1544''E | **2018** |
|  | CRS-47 | Chakia, Chandauli, Uttar Pradesh (India) | 25°2'57.1632''N 83°13'16.1544''E | **2016** |
|  | CRS-50 | Kadhikueya, Payagpur, Baharaiech, Uttar Pradesh (India) | 27°24'0.3708''N 81°46'33.3804''E | **2016** |
|  | CRS-54 | Kadhikueya, Payagpur, Baharaiech, Uttar Pradesh (India) | 27°24'0.3708''N 81°46'33.3804''E | **2017** |
|  | CRS-55 | Kadhikueya, Payagpur, Baharaiech, Uttar Pradesh (India) | 27°24'0.3708''N 81°46'33.3804''E | **2018** |
|  | CRS-57 | Kadhikueya, Payagpur, Baharaiech, Uttar Pradesh (India) | 27°24'0.3708''N 81°46'33.3804''E | **2016** |
|  | CRS-77 | Akabarpur, Ambedkar Nagar, Uttar Pradesh (India) | 26°25'37.1532''N 82°31'59.2032''E | **2018** |

**Supplementary Table 2.** Oligonucleotide primer sequences used for SYBR Green real time RT-PCR expression analysis

| S.No. | Gene symbol | Forward Primer | Reverse Primer |
| --- | --- | --- | --- |
|  | *TaZIP1* | GTCCCCCTACTTCTACCGCT | TGGTTGACCCTCTGCCTGTT |
|  | *TaZIP3* | GGGAAATGGAGAACYCCTGGATG | GGCATAGAGATCTTGAAAGCAATTGC |
|  | *TaZIP5* | AGGTTTCGCCTCAAGTCTGTCTTG | GGCTATTCTCGTCGTAAGCAGAG |
|  | *TaZIP6* | GTCATCATCTCTGAAACTGAAGAAGG | CCCTCTATACATTTCACTATGRCC |
|  | *TaZIP7* | ACAGGCAGTATGTTSGGACGTAG | CAGCAAGTGATGGCCTATGTCG |
|  | *TaZIP10* | GTGGATCTCATTGCTGCTGA | AGCCCAAATAGCCAGTGATG |
|  | *TaZIP10-like1* | GCCTTCTTCTTTGCCATCAC | GGGCTGTATGGGTCGTAGAA |
|  | *TaZIP13* | CGCAAGCSTACAACATGAAACAGT | CTTYAGACACGCTACTGGGTTGG |
|  | *TaZIP13* | CGCGAGCCTACAACTTGAAACAG | CTTYAGACACGCTACTGGGTTGG |
|  | *TaZIP15* | CTCTCTGCGCTGGTTGCTTT | TGGGAGGACTCCGGCAACAG |
|  | *TaActin3* | GACGCACAACAGGTATCGTGTTG | CAGCGAGGTCAAGACGAAGGATG |
|  | *TaSuccDH* | TTTGCTCTCCGTGGTGCCTTTGG | GAAGATGTGTAGCTCCTTGCTTGC |

**Supplementary Table 3.** Plant growth promoting traits of zinc solubilizing rhizobacterial strains

| Rhizobacterial strains | P-solubilization | K-solubilization | IAA production (μg ml^-1^) | ACC deaminase activity | Siderophore  production | Ammonia production |
| --- | --- | --- | --- | --- | --- | --- |
| *Bacillus* *glycinifermentans* CRS-9 | +++ | ++ | 6.61 | - | - | +++ |
| *Microbacterium* *oxydans* CRS-17 | +++ | - | 6.43 | - | +++ | - |
| *Paenarthrobacter* *nicotinovorans* CRS-30 | ++ | +++ | 17.16 | - | ++ | - |
| *Bacillus* *tequilensis* CRS-38 | +++ | ++ | 8.61 | ++ | ++ | ++ |

+: Positive; - Negative

**Supplementary Table 4:** Morphological and biochemical characterization of zinc solubilising rhizobacterial strains

| Culture characteristics | **Rhizobacterial strains** | | | |
| --- | --- | --- | --- | --- |
|  | **CRS9** | **CRS-17** | **CRS-30** | **CRS-38** |
| **Morphological** | | | | |
| Colony | Translucent, irregular, undulate, smooth, medium and flat colony | Creamy white, irregular, raised, medium, rough and opaque colony | Creamy white, circular, entire, pointed, smooth and raisedcolony | Creamy white, irregular, raised, medium, rough and opaque colony |
| Cell shape | Rod | Rod | Rod | Rod |
| Gram reaction | +Ve | +Ve | +Ve | +Ve |
| Endospore | +Ve | +Ve | NA | +Ve |
| **Biochemical** |  |  |  |  |
| Catalase | **+** | **+** | **+** | **+** |
| Oxidase | **-** | **-** | **-** | **-** |
| Nitrate | **+** | **-** | **-** | **+** |
| H_2_S | **-** | **+** | **-** | **+** |
| Citrate utilization | **+** | **+** | **+** | **-** |
| Voges Proskauer's | **-** | **-** | **-** | **-** |
| Esculin hydrolysis | **+** | **+** | **+** | **+** |
| Methyl red | **+** | **-** | **-** | **+** |
| Indole | **-** | **-** | **-** | **-** |
| ONPG | **-** | **-** | **-** | **-** |
| Lysine | **+** | **+** | **+** | **+** |
| Ornithine | **+** | **+** | **+** | **+** |
| Urease | **+** | **-** | **+** | **+** |
| Phenylalanine | **-** | **-** | **-** | **-** |
| Malonate | **+** | **-** | **-** | **-** |
| Arabinose | **-** | **-** | **-** | **-** |
| Xylose | **+** | **-** | **+** | **-** |
| Adonitol | **-** | **-** | **-** | **-** |
| Rhamnose | **-** | **-** | **-** | **+** |
| Cellobiose | **-** | **-** | **-** | **-** |
| Melibiose | **-** | **-** | **-** | **+** |
| Saccharose | **-** | **+** | **+** | **-** |
| Raffinose | **-** | **-** | **-** | **-** |
| Trehalose | **-** | **+** | **+** | **-** |
| Glucose | **+** | **+** | **+** | **+** |
| Lactose | **+** | **-** | **-** | **-** |

*** +** indicate the activity; **-** indicate the no activity

**Table 5.** Details of GenBank accession number of zinc solubilising rhizobacterial strains with their nearest matching strain.

| Strain | Accession Number | Identified as | Sequence Length | Completeness (%) | E Value | Similarity (%) | Nearest match | NAIMCC Accession No. |
| --- | --- | --- | --- | --- | --- | --- | --- | --- |
| CRS-9 | MH497203 | *Bacillus glycinifermentans* | 1,412 | 100 | 0.0 | 99.5 | *Bacillus glycinifermentans* strain JTYP3 (MH475943) | NAIMCC-B-02249 |
| CRS-17 | MH497204 | *Microbacterium oxydans* | 1,368 | 100 | 0.0 | 100 | *Microbacterium oxydans* strain DSM 20578 (NR_044931) | NAIMCC-B-02517 |
| CRS-30 | MH497212 | *Paenarthrobacter nicotinovorans* | 1,378 | 100 | 0.0 | 99.1 | *Paenarthrobacter nicotinovorans* strain DSM 420 (NR_026194) | NAIMCC-B-02251 |
| CRS-38 | MH497217 | *Bacillus tequilensis* | 1,407 | 100 | 0.0 | 99.7 | *Bacillus tequilensis* strain 10b (NR_104919) | NAIMCC-B-02516 |

**Supplementary Fig. 1.** Plate showing Zn solubilization by *Bacillus glycinifermentans* CRS-9, *Microbacterium oxydans* CRS-17, *Paenarthrobacter nicotinovorans* CRS-30 and *Bacillus tequilensis* CRS-38 on tris-minimal medium supplemented with zinc phosphate at 28 °C after 7 days of inoculation.

| **(A)** *Bacillus glycinifermentans* CRS-9**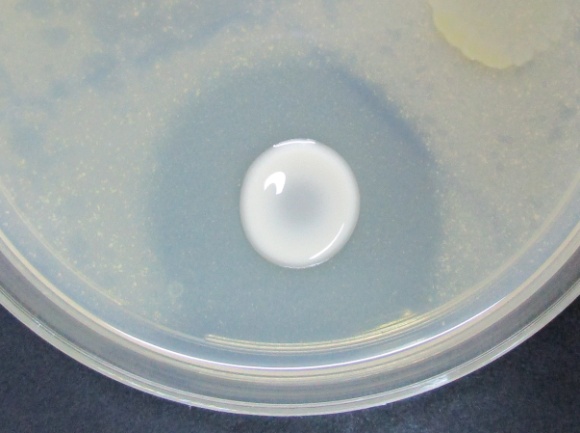** | **(B)** *Microbacterium oxydans* CRS-17**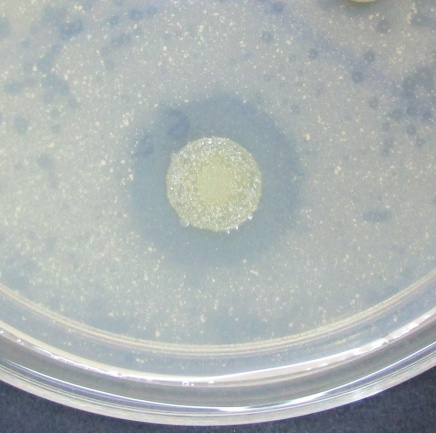** |
| --- | --- |
| **(C)** *Paenarthrobacter nicotinovorans* CRS-30  **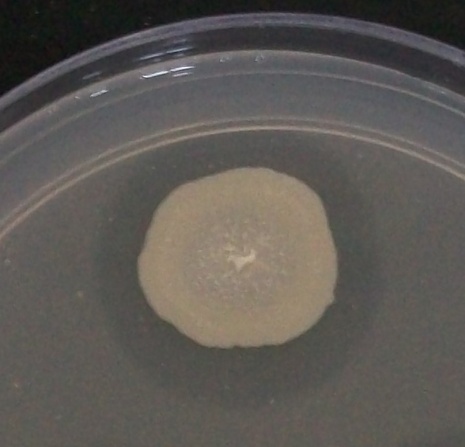** | **(D)** *Bacillus tequilensis* CRS-38 **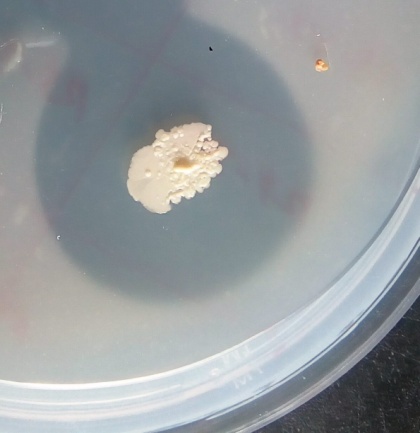** |
| **(E)** Negative control**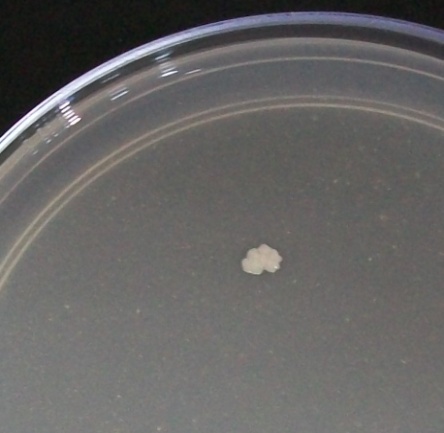** |  |

**Supplementary Fig. 2.** Effects of seed biopriming on plant growth and seedling vigour at 30 days after sowing under glasshouse conditions.

| (A) Absolute control  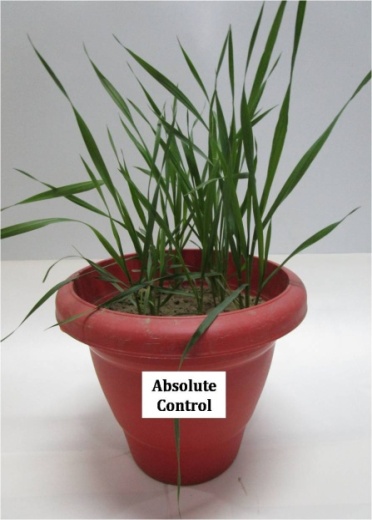 | (B) *Bacillus glycinifermentans* CRS-9  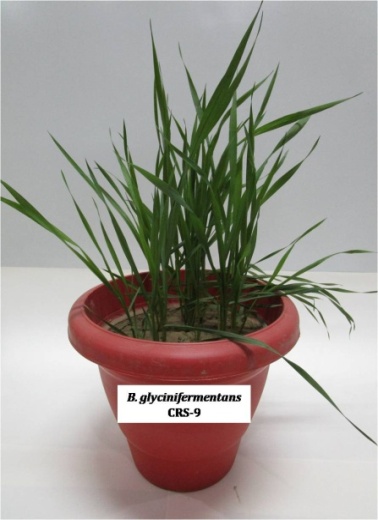 |
| --- | --- |
| (C) *Microbacterium oxydans* CRS-17  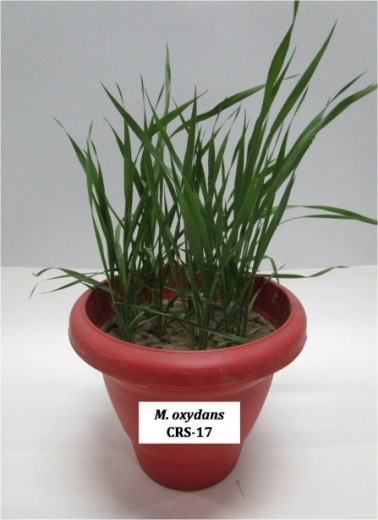 | (D) *Paenarthrobacter nicotinovorans* CRS-30  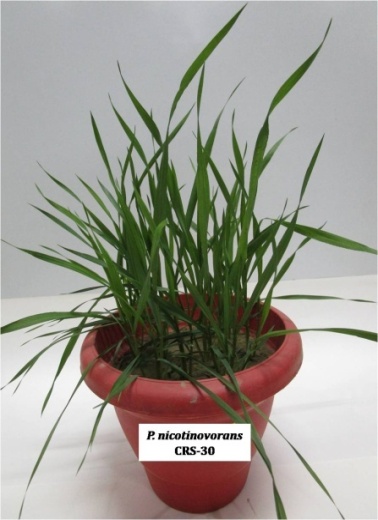 |
| (E) *Bacillus tequilensis* CRS-38  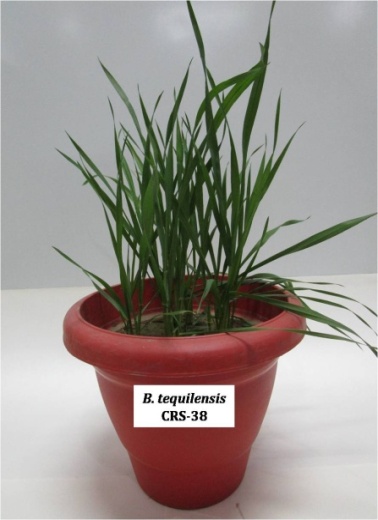 |  |
